# Dichotomy in FcγRIIB deficiency and autoimmune-prone SLAM haplotype clarifies the roles of the Fc receptor in development of autoantibodies and glomerulonephritis

**DOI:** 10.1186/s12865-014-0047-y

**Published:** 2014-10-24

**Authors:** Yasuyoshi Kanari, Akiko Sugahara–Tobinai, Haruka Takahashi, Masanori Inui, Akira Nakamura, Sachiko Hirose, Toshiyuki Takai

**Affiliations:** Department of Experimental Immunology and CREST Program of JST, Institute of Development, Aging and Cancer, Tohoku University, 4-1 Seiryo, Sendai, 980-8575 Japan; Department of Immunology, Kanazawa Medical University, Kanazawa, Ishikawa 920-0293 Japan; Department of Pathology, Juntendo University School of Medicine, 2-1-1 Hongo, Bunkyo-ku, Tokyo 113-8421 Japan

**Keywords:** Autoimmune disease, Systemic lupus erythematosus, Autoantibody production, B cells, Myeloid cells, Inhibitory receptor

## Abstract

**Background:**

The significance of a unique inhibitory Fc receptor for IgG, FcγRIIB (RIIB), in the prevention of spontaneous production of autoantibodies remains controversial, due mainly to the fact that the *RIIB* locus is adjacent to the autoimmune-related *SLAM* locus harboring the genes coding for signaling lymphocyte activation molecules, making it difficult to isolate the effect of RIIB deletion from that of *SLAM* in gene-targeted mice. Our objective was to determine the influence of *RIIB* deletion on the spontaneous development of autoimmune diseases and to compare it with that of potentially pathogenic *SLAM*.

**Results:**

We established two congenic C57BL/6 (B6) strains, one with the *RIIB* deletion and the other with *SLAM*, by backcrossing 129/SvJ-based *RIIB*-deficient mice into the B6 genetic background extensively. The RIIB deficiency indeed led to the production and/or accumulation of a small amount of anti-nuclear autoantibodies (ANAs) and to weak IgG immune-complex deposition in glomeruli without any obvious manifestation of lupus nephritis. In contrast, pathogenic *SLAM* in the B6 genetic background induced ANAs but no IgG immune-complex deposition in the kidneys. Naïve *SLAM* mice but not RIIB-deficient mice exhibited hyperplasia of splenic germinal centers.

**Conclusion:**

The present results clarify the roles of RIIB in preventing production and/or accumulation of a small amount of ANAs, and development of glomerulonephritis. The combined effects of RIIB deletion and pathogenic SLAM can lead to severe lupus nephritis in the B6 genetic background.

**Electronic supplementary material:**

The online version of this article (doi:10.1186/s12865-014-0047-y) contains supplementary material, which is available to authorized users.

## Background

Systemic lupus erythematosus (SLE) is characterized by spontaneous production of autoantibodies including anti-nuclear autoantibodies (ANAs), and by development of vasculitis and autoimmune glomerulonephritis (so-called lupus nephritis) preferentially in females. The genetic susceptibility loci have been mapped throughout the chromosomes, the major locus having been mapped to the chromosome 1 telomeric region, in which many immunoregulatory genes are concentrated in humans as well as in mice [1-3]. One of the major susceptible haplotypes in this region has been identified in murine SLE models as the group of seven genes for the signal lymphocyte activation molecule (SLAM or CD2) family members, which regulate cognate interactions between T and B cells, and signaling [4]. Interestingly, while both SLAM haplotypes 1 and 2 in non-autoimmune strains of mice, such as C57BL/6 (B6) and 129/SvJ, respectively, are not harmful when expressed in the original genetic backgrounds of the host strains, SLAM haplotype 2 in 129 mice (*SLAM*^129^) renders the hosts susceptible to autoimmunity, ANAs being produced when expressed in the B6 genetic background [4,5]. Interestingly, four members of the SLAM family cluster, *Cd48*, *Ly108*, *Cd84*, and *Cs1*, vary in their expression in splenic B and/or T cells between B6 mice harboring haplotypes 1 and 2 [4]. In particular, Ly108 was suggested to be one of the determinants of the autoimmunity susceptibility [4,6].

Another important susceptible gene for autoimmunity in this 1q telomeric region has been suggested to be the gene coding for a unique inhibitory Fc receptor for IgG (FcγR), FcγRIIB (RIIB) [7-11]. Generally, it is well established that the FcγR family molecules expressed on myeloid-lineage cells and B cells play crucial roles in adaptive immune responses and inflammation, in which activating-type FcγRs such as FcγRIII initiate and accelerate the responses, while inhibitory RIIB decreases them upon co-engagement with other activating-type FcγRs or B cell antigen receptors via IgG immune complexes (IgG-ICs) [7,9-12]. Thus, RIIB executes important IgG-mediated feedback regulation of cell activation, proliferation and antibody/cytokine production, as demonstrated in different experimental settings using RIIB-deficient (*RIIB*^*−*/−^) mice [13-15]. In contrast to this well-established feedback regulation, the roles of RIIB in maintaining tolerance and in preventing spontaneous development of autoimmunity, however, remain uncertain due to the inconsistent phenotypes observed in *RIIB*^*−*/−^ mice by different research groups, particularly in terms of the level of ANAs and the severity of lupus nephritis [8,16-18]. In addition, a recent report [19] pointed out the role of RIIB expressed in liver sinusoidal endothelial cells in the clearance of small size IgG-ICs, so the additional RIIB function of minimizing the level of pathologic IgG-ICs in the blood should also be taken into account.

Importantly, the *FcR* locus is adjacent to the *SLAM* locus in the 1q telomere of the mouse genome [1,4]. It has been pointed out that special care should be taken when examining the precise influence of any targeted gene, such as that of RIIB, located in this region on the occurrence of spontaneous autoantibody production and of lupus nephritis, by eliminating any influence of SLAM members in gene-targeted mice generated with 129-derived ES cells and backcrossed into the genetic background of B6 [1,4,5]. Therefore, the differences in the observed phenotypes in *RIIB*^*−*/−^ mice could be due, at least in part, to the heterogeneity of the genomic interval around the *RIIB*^*−*/−^ locus in the B6 background, which might be co-segregated with the autoimmune-prone *SLAM*^129^ haplotype discussed above [4,6]. In addition to the genetic heterogeneity of *RIIB*^*−*/−^ mice, unidentified differences in environmental conditions such as those in nutrition and the gut microbial flora should also be considered.

To clarify the role of RIIB in peripheral tolerance in the absence of any effect of the pathogenic *SLAM*^129^ haplotype, we separated the *RIIB*^*−*/−^ locus and the *SLAM*^129^ locus by extensively backcrossing our *RIIB*^*−*/−^ mice into the B6 background, followed by analysis of microsatellite markers in this genetic interval in the offspring. Each of the two congenic B6 strains obtained, one harboring *RIIB*^*−*/−^ and the other pathogenic *SLAM*^129^, exhibited unique phenotypes as to germinal center formation in the spleen, ANAs in serum, IgG-IC deposition in the kidneys, and development of lupus nephritis, which revealed the exact roles of RIIB and *SLAM*^129^-derived set of molecules in maintaining tolerance, which were examined in detail in this study.

## Methods

### Mice

The original FcγRIIB (RIIB)-deficient mice constructed with a 129 and B6 hybrid background [13] were backcrossed into the B6 background for 12 generations as described previously [16] using C57BL/6NCrj mice (Charles River Inc. Japan, Kanagawa, Japan), and then, in this study, they were further backcrossed up to the 28th generation (Additional file 1: Figure S1A). The offspring were genotyped by analysis of simple sequence length polymorphisms (SSLP) using the seventeen SSLP markers on chromosome 1, which allow discrimination of the polymorphisms in B6 and 129 mice, particularly around the *Fcgr2b* gene and for the *SLAM* haplotype (Additional file 1: Figure S1). SSLP analysis was conducted by the Central Institute of Experimental Animals (Kawasaki, Japan). We established two substrains, N22 and N28 B6.*RIIB*^−/−^*SLAM*^B6^ (*RIIB*^−/−^) and B6.*RIIB*^+/+^*SLAM*^129^ (*SLAM*^129^) mice as well as N28 B6.*RIIB*^−/−^*SLAM*^129^ (*RIIB*^−/−^*SLAM*^129^) mice as controls (Additional file 1: Figure S1). RIIB-deficient mice were also provided by S. Hirose of Juntendo Univ. (Tokyo), or purchased from TACONIC® Farms (Germantown, NY, USA). When pertinent, we refer to our N28 *RIIB*^−/−^ mice used in the present study as *RIIB*^−/−^SENDAI and those obtained or purchased from others as *RIIB*^−/−^TOKYO or *RIIB*^−/−^TACONIC, respectively, in order to distinguish the data in figures. In several experiments, we also employed B6 mice obtained from CLEA Japan, Inc. (Tokyo, Japan) as aged controls or a 12-month-old NZB/W F_1_ female mouse from Japan SLC, Inc. (Shizuoka, Japan) as an autoimmune control. For adaptation, the B6 mice of non-littermate controls and NZB/W F_1_, *RIIB*^−/−^TOKYO, and *RIIB*^−/−^TACONIC mice were housed in our facility for at least 6 months before use. All mice were housed in the Experimental Animal Facility of The Institute of Development, Aging and Cancer (Tohoku University, Sendai, Japan), an environmentally controlled and specific pathogen-free facility. All animal protocols were reviewed and approved by the Animal Studies Committee of the Tohoku University, and we followed the guidelines defined by the committee.

### Genotyping of SNPs

For isolation of genomic DNA samples, tail tips from mice were lysed by incubation in 100 μl of tail lysis buffer [50 mM Tris-HCl (pH8.0), 20 mM NaCl, 1 mM EDTA, 0.063% SDS and 1 mg/ml proteinase K] for at least 4 hours at 55°C. Tail lysates were diluted with 10 volumes of water, and then subjected to PCR amplification using gene-specific PCR primer pairs (Additional file 1: Table S1) and KOD FX polymerase (TOYOBO Co. Ltd., Osaka, Japan) as follows: initial 2-min incubation at 94°C, 30 cycles consisting of 95°C for 30 s, 60°C for 30 s and 72°C for 90 s, and a final 10 min elongation step at 72°C. For determination of SNPs in the vicinity of the *Fcgr2b* locus, PCR products were directly sequenced using specific primers (Additional file 1: Table S1) with a 3130x1 Sequencer (Life Technologies, Waltham, MA, USA).

### Antibodies

For FACS analysis, the following fluorochrome-labeled antibodies specific for mouse molecules were used: CD4-FITC, CD4-Alexa647, CD19-Pacific blue (BioLegend, Inc., San Diego, CA, USA), and GL7-FITC and FAS-PE (Becton Dickinson Ltd., Franklin Lakes, NJ, USA). For immunohistochemistry, anti-mouse-IgM-Alexa488, anti-rat-IgG-Alexa488, and anti-mouse-IgG-Alexa555 (Invitrogen Life Technologies, Carlsbad, CA, USA), anti-mouse-IgD-biotin (BioLegend), GL7-Alexa488 (eBiosciences Inc., San Diego, CA, USA), and anti-mouse IgM F(ab’)_2_ Alexa647 (Jackson ImmunoResearch laboratories Inc., West grove, PA, USA) were used.

### Histological study and immunohistochemistry

Kidneys were fixed with 4% paraformaldehyde in phosphate buffer. Specimens were embedded in paraffin, sectioned at 2 μm, and then stained with hematoxylin and eosin (HE) or periodic acid-Schiff (PAS). To evaluate glomerular lesions, at least 25 glomeruli were examined per HE-stained section by light microscopy based on pathological manifestations of inflammation, and/or tissue damage in a blind manner [20]. The severity of glomerulonephritis was estimated as follows; grade 0, normal; grade 1, neutrophil infiltration and segmental mesangial proliferation; grade 2, limited lobulated glomeruli in grade 1; and grade 3, crescent formation and severe lobulated glomeruli with lymphocyte infiltration (Additional file 1: Figure S2). To assay IgG-ICs deposition, kidney samples were embedded in O.C.T. compound (Tissue-Tek, Miles, Inc., Elkhart, IN), snap-frozen in liquid nitrogen, and then sectioned at 5 μm. The sections were fixed with 4% paraformaldehyde for 20 min, washed again three times with PBS, and then preincubated for 1 hour at room temperature with PBS containing 20% BSA to block nonspecific antibody binding. They were then incubated overnight at room temperature with affinity-purified Alexa488-conjugated goat F(ab’)_2_ fragments (H + L chain) anti-mouse IgG (Invitrogen). After washing three times with PBS, slides were mounted and examined under an Olympus BX50 microscope equipped with an Olympus BH2-RFL-T3 mercury lamp and appropriate optics.

Spleens were embedded in O.C.T. compound, frozen in liquid nitrogen, and then sectioned at 8 μm. Sections were fixed with acetone for 20 min at −20°C and then air-dried for 1 hour. After washing with PBS six times, sections were pre-incubated for 1 hour at room temperature with PBS containing 2% BSA for blocking, and then incubated with Alexa488-labeled anti-mouse IgM (Invitrogen) or Alexa488-labeled GL7, and biotinylated anti-mouse IgD (eBiosciences) for staining of splenic B cells. After washing with PBS three times, sections were incubated with 1:2000 diluted Alexa546-conjugated streptavidin at 4°C for 30 min.

### Determination of ANA levels in sera from indirect immunofluorescence and by HEp2 cell staining combined with ELISA

For determination of gross ANA levels, serum samples from 24–48-wk-old mice were serially diluted from 1:40 to 1:320 with PBS containing 1% BSA. HEp-2 cell-seeded slides (Fluoro HEPANA test; MBL, Nagoya, Japan) were incubated with diluted serum samples for 45 min at room temperature, followed by brief washing and incubation with PBS for 60 min. Anti-nuclear antibodies (ANA) were detected with Alexa488-labeled anti-mouse IgG F(ab’)_2_ (Invitrogen Life Technologies) and visualized under a fluorescence microscope (Olympus, Tokyo, Japan). To determine ANA levels and the IgG isotypes by ELISA, sera from mice were diluted 1:100 with PBS containing 1% BSA and then added to a HEp-2 coated 96-well plate (ANA HEp Screen ELISA kit; Abnova, Taipei, Taiwan). ANA and the IgG isotypes were detected with HRP-conjugated goat anti-mouse IgG Fc fragment and anti-mouse IgG itsotype-specific antibodies (Bethyl Laboratory Inc., TX, USA), respectively.

### ELISA measurement of Igs and anti-DNA antibodies

The serum levels of total IgM, IgG, and autoantibodies were measured by ELISA. For anti-ssDNA and -dsDNA antibodies, high-bound flat-bottom 96-well plates were coated firstly with 0.001% protamine sulfate in water for 60 min, and then with 5 μg/ml of single-strand or double-strand calf thymus DNA (SIGMA) diluted in 0.015 M sodium citrate containing 0.15 M NaCl for 90 min, followed by blocking with 50% FCS in PBS for 60 min. Serum samples were diluted to 1:100 with PBS containing 1% BSA and 0.05% Tween 20, and then applied to the DNA-coated 96-well plates. Serum from a 12-month-old NZB/W F_1_ female mouse was used as a control. After washing three times with PBS containing 0.05% of Tween 20, ANAs were detected with anti-mouse IgG conjugated with HRP as detection antibodies. The total IgM and IgG levels were measured with a Mouse IgM ELISA Quantitation Set and a Mouse IgG ELISA Quantitation Set (Bethyl Laboratory, Inc.), respectively, according to the manufacturer’s protocols. To titrate anti-DNA antibodies, a positive control serum from an old female NZB/W F_1_ mouse was examined for the anti-dsDNA and anti-ssDNA levels using mouse monoclonal anti-dsDNA IgG2a (Abcam, Cambridge, UK), which is also cross-reactive with ssDNA on ELISA.

### Flow cytometry

Spleens were isolated from B6 and *RIIB*^−⁄−^ mice, and suspended as single cells with PBS containing 1% BSA. Red blood cells were depleted by lysis with 0.144 M NH_4_Cl. 2.0 × 10^6^ cells per sample were blocked with 25 μg/ml anti-FcγRII/III (2.4G2), incubated for 15 min at 4°C, and then stained with fluorochrome-labeled antibodies. All fluorescent antibodies were used at 1:200 dilution. Data were collected with FACSCalibur and FACSAria, and analyzed using CellQuest and FACSDiva software (Becton Dickinson Ltd.), respectively.

### Statistical analysis

Statistical analysis was performed using Microsoft Excel for Mac 2011 software version 14.2.3 (Microsoft Corp., Seattle, WA) or Prism6 software (Graph Pad Software Inc., La Jolla, CA). Data are displayed when appropriate as means ± SD. Data were compared for statistical differences using Student’s *t* test with two-tailed analysis, Log-rank test, or Chi-square test, as indicated in each figure legend. *P* values are shown in the relevant figures. *P* <0.05 was considered as statistically significant.

## Results

### *RIIB*^−/−^, *SLAM*^129^, and *RIIB*^−/−^*SLAM*^129^ lines with the B6 background thrive normally

**Figure 1:**
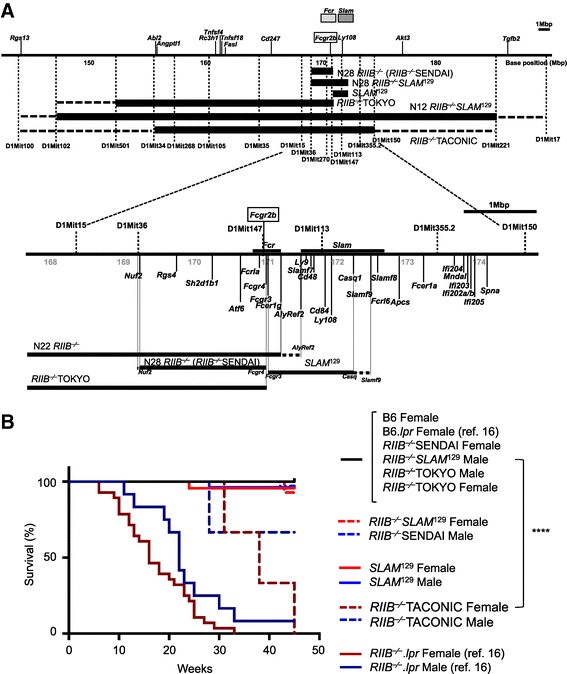
**Chromosomal configurations in the vicinity of the** ***Fcgr2b*** **gene and** ***SLAM*** **locus of congenic mouse lines. (A)** Physical mapping was compiled according to the Ensemble Genome Browser (http://www.ensembl.org/). *Upper*, gross schematic view, and *Lower*, detailed genomic structure with SSLP markers, and the genes related to the immune system and examined by SNP analysis (Additional file 1: Table S1 and Additional file 1: Figure S1). Scale bar =1 Mbp. Thick horizontal bars below the map indicate 129 strain-derived intervals, and the flanking, thin broken lines denote intervals unidentified for the B6 or 129 strain. Other areas not shown were derived from B6. The *RIIB* ^−⁄−^TACONIC line had a 129-derived interval from *D1Mit34* to *D1Mit150* at the minimum, while our N12 *RIIB* ^−⁄−^ had an interval from *D1Mit102* to *D1Mit221* at the minimum, according to our analysis. The *Fcr* and *SLAM* loci are shown as shaded boxes at the top of the *Upper* panel or as thick lines above the map in the *Lower* panel. The *Fcgr2b* gene is boxed. When pertinent, we refer to our N28 *RIIB* ^−/−^ mice used in the present study as *RIIB* ^−/−^SENDAI and those obtained or purchased from others as *RIIB* ^−/−^TOKYO or *RIIB* ^−/−^TACONIC, respectively, in order to distinguish the data in figures. **(B)** Survival curves for B6 (female, *n* =17), *RIIB* ^−/−^SENDAI (female, *n* =30; male, *n* =35), *SLAM* ^129^ (female, *n* =23; male, *n* =27), *RIIB* ^−/−^ *SLAM* ^129^ (female, *n* =14; male, *n* =18), *RIIB* ^−/−^TOKYO (female, *n* =7; male, *n* =4), and *RIIB* ^−/−^TACONIC (female, *n* =3; male, *n* =3) mice. Mice of different lines were examined as to their survival until week 45. For comparison, survival curves for B6.*lpr* (female, *n* =17) and *RIIB* ^−⁄−^ *lpr* (female, *n* =28; male, *n* =12) from our previous study [16] are superimposed. ****Significantly different between female *RIIB* ^−/−^TACONIC *vs* female *RIIB* ^−/−^SENDAI (*P* <0.0001, Log-rank test).

*RIIB*^−/−^ and *SLAM*^129^ mice were generated by backcrossing our 12th-backcross (N12) *RIIB*^*−*/−^ mice [13,16] into the B6 genetic background further to the 28th generation (N28). Analysis of a series of microsatellite markers including *D1Mit15*, *36*, and *113* in the vicinities of the *Fcgr2b* and *SLAM* loci revealed the successful separation of the *RIIB*^*−*/−^ locus and the *SLAM*^129^ locus during the backcross (Figure 1A; for details, see Additional file 1: Figure S1 and Additional file 1: Table S1). We also obtained a mouse line with the combined loci, N28 *RIIB*^*−*/−^*SLAM*^129^, as a control (Figure 1A and Additional file 1: Figure S1). Both the N28 *RIIB*^−/−^ (*RIIB*^−/−^SENDAI) and N18 *SLAM*^129^ mice thrived normally, at least up to 45 weeks after birth, and the *RIIB*^−/−^*SLAM*^129^ mice did as well despite the fact that female *RIIB*^−/−^*SLAM*^129^ mice manifested obvious glomerulonephritis, as judged on histology (Figure 1B, see below), which was consistent with our previous observation for N12 *RIIB*^−/−^ mice [16]. Four (3 females and 1 male) out of six (3 of each gender) *RIIB*^−/−^ mice obtained from a breeder (*RIIB*^−⁄−^TACONIC) died before 45 weeks of age in our facility, due probably to lupus nephritis [21] (Figure 1B). Mice obtained from another facility (*RIIB*^−⁄−^TOKYO) [18] thrived like our *RIIB*^−/−^ mice did. It should be stressed that our *RIIB*^−/−^SENDAI line and *RIIB*^−/−^ animals from other sources were derived from a common origin [13], and backcrossed into the B6 background at different facilities. These results indicate that, even under identical environmental conditions, FcγRIIB-deficient mice with 129-derived genetic intervals of different lengths around the *RIIB*^−/−^ locus show different mortalities, raising the possibility of some influence(s) of unidentified genetic factor(s) within the intervals in the B6 genetic background. Also, the separation of the *RIIB*^−/−^ locus from that of autoimmune-prone *SLAM*^129^ as well as extensive shortening of the 129-derived interval (*Nuf2* to *Fcgr4*, Figure 1A) allowed the establishment of N28 *RIIB*^−/−^SENDAI mice with a normal survival rate, which we mainly used in our analysis described below.

### *RIIB*^−/−^ mice produce a small amount of ANAs

**Figure 2:**
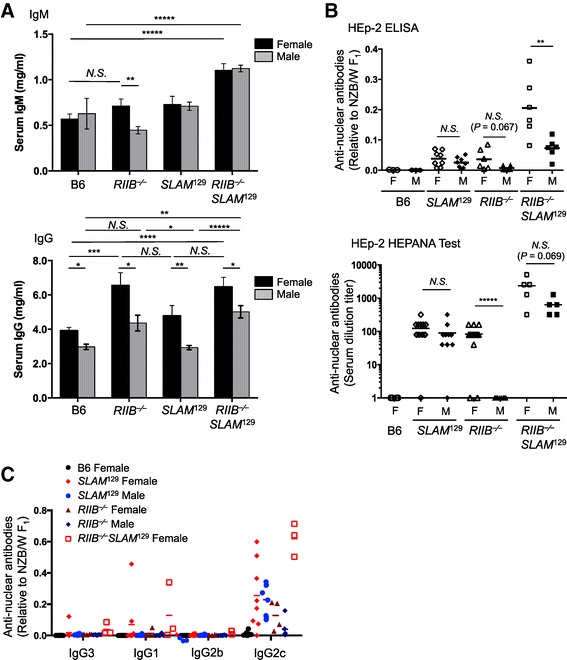
**Female-biased production of anti-nuclear antibodies in** ***RIIB*** ^**−/−**^ **mice. (A)** The total IgM and IgG levels in sera from different lines, including B6, *RIIB* ^−/−^, *SLAM* ^129^, and *RIIB* ^−/−^ *SLAM* ^129^, at 24–28 weeks of age of both genders were measured by ELISA. For IgM and IgG determinations in male B6 mice, *n* =3 and 5, respectively; for other determinations, *n* ≥8. The data in each panel are from two separate measurements and are presented as means ± S.D. *P <0.05; **P <0.01; ***P <0.001; ****P <0.0001; *N.S.*, not significant. Student’s *t*-test. **(B)** Spontaneous production of ANAs by *SLAM* ^129^ (*n* =16), *RIIB* ^−/−^ (*n* =12), *RIIB* ^−/−^ *SLAM* ^129^ (*n* =12), and B6 (*n* =6) mice at 36 weeks (*Upper*) or 24 weeks (*Lower*) of age of both genders was measured in serum samples by HEp-2 staining combined with ELISA (*Upper*) and HEp-2-HEPANA Test (*Lower*), and expressed as the levels relative to that in sera collected from a 12-month-old female NZB/W F_1_ mouse, whose anti-dsDNA and anti-ssDNA levels were 6.64 μg/ml and 4.69 μg/ml, respectively. Horizontal lines represent the mean values. **P <0.01; ****P <0.0001; *N.S.*, not significant. Student’s *t*-test. **(C)** IgG isotypes of ANAs in sera from *RIIB* ^−/−^ and *SLAM* ^129^ mice of both genders. ANA IgG isotypes were determined by HEp-2 staining combined with ELISA, and presented as values relative to that in a 12-month-old NZB/W F_1_ control female mouse, as described in B.

To gain an insight into the difference between *RIIB*^−/−^ and S*LAM*^129^ mice, and its relation to autoimmunity, we measured the total IgM and IgG levels in sera from these locus-separated lines and the combined line, *RIIB*^−/−^S*LAM*^129^, as a control, at the age of 24–28 weeks. We found that the IgM levels in *RIIB*^−/−^S*LAM*^129^ mice but not *RIIB*^−/−^ or S*LAM*^129^ mice were higher than that in B6 mice, while the IgG levels were higher in female *RIIB*^−/−^ mice and *RIIB*^−/−^S*LAM*^129^ mice of both genders, but not S*LAM*^129^ mice, than that in B6 mice (Figure 2A). Regarding autoantibodies, both *RIIB*^−/−^ and *SLAM*^129^ mice at 36 weeks of age produced a detectable level of ANAs, but in much smaller amounts than *RIIB*^−/−^*SLAM*^129^ mice did, as shown on immunofluorescent staining of HEp-2 cells combined with ELISA, and the levels were less than one-tenth to that in a 12-month-old NZB/W F_1_ positive control mouse (Figure 2B, *upper*). There was a tendency that the ANA production and/or accumulation was more pronounced in female than male *RIIB*^−/−^ mice (*P* =0.067; Figure 2B, *upper*), the female bias being more evident on the titration with HEp-2-HEPANA Test at 24 weeks of age (*P* <0.0001; Figure 2B, *lower*). This gender bias was not clear in *SLAM*^129^ mice or a breeder’s *RIIB*^−/−^ line (Figure 2B). We also checked the IgG subclass of ANAs by HEp-2 ELISA (Figure 2C), and found that IgG2c was the most prominently produced subclass of ANAs in *RIIB*^−/−^ mice, suggesting that type 1 helper T cells are dominantly activated.

**Figure 3:**
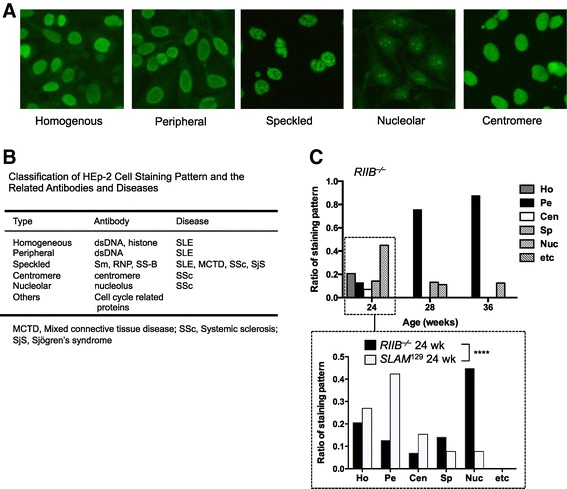
**Qualitative analysis of ANAs in** ***RIIB*** ^**−⁄−**^ **and** ***SLAM*** ^**129**^ **mice. (A)** Classification of staining profiles of HEp-2 cells. Representative staining patterns observed for serum samples from *RIIB* ^−⁄−^ *SLAM* ^129^ mice are shown as fluorescently stained cells. **(B)** The classification of HEp-2 cell staining pattern and the related antibodies and diseases. (**C**, *Upper*) Comparison of the HEp-2 staining patterns of female *RIIB* ^−/−^ mice at different ages. The staining patterns were divided into six categories as shown in **(B)**. (**C**, *Lower*) Comparison of the staining patterns between female *RIIB* ^−/−^ and *SLAM* ^129^ mice at 24 weeks of age. For classification, staining profiles in sera from ≥13 mice were evaluated. Ho, homogeneous; Pe, peripheral; Cen, centromere; Sp, speckled; Nuc, nucleolar; etc, others. Significantly different between *RIIB* ^−/−^ and *SLAM* ^129^ (*P* <0.0001 by Chi-square test).

Examination of the staining profile of HEp-2 cells (Figure 3A, B) indicated that ANAs detected in sera from female *RIIB*^−/−^ mice at 24 weeks of age comprised a mixture of anti-DNA, histone and nucleolus antibodies, and others, because the staining could be classified into nucleolar, homogeneous, and other types, while for the ANAs at 28 and 36 weeks of age peripheral-type staining was dominant, indicating the anti-dsDNA antibodies were major (Figure 3C). When we compared the staining profiles in female *RIIB*^−/−^ and *SLAM*^129^ mice at 24 weeks of age, we observed that the peripheral-type staining was dominant in *SLAM*^129^ mice (Figure 3C, *lower*), suggesting that the compositions of ANAs produced by *RIIB*^−/−^ and *SLAM*^129^ mice at 24 weeks of age are qualitatively different, as determined based on the HEp-2 cell staining profiles, albeit the assay used is not sufficiently quantitative.

**Figure 4:**
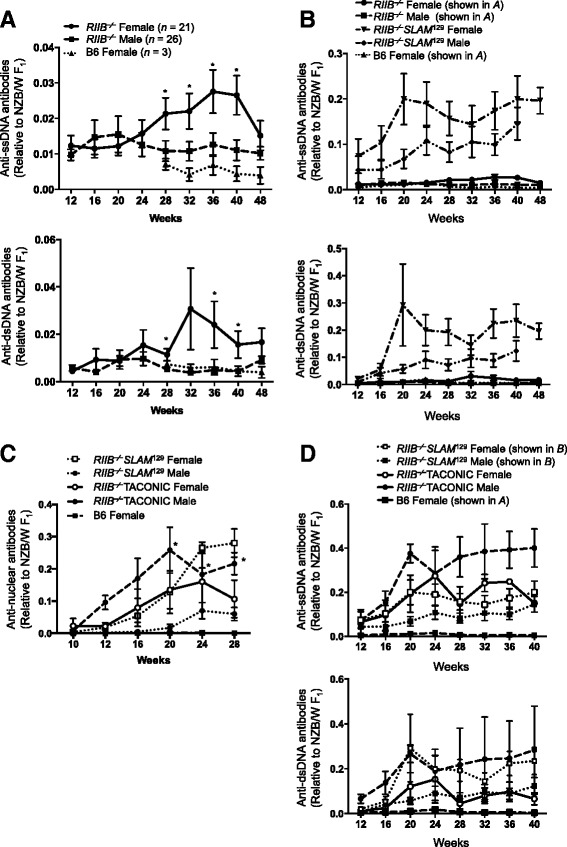
**Increase of anti-DNA antibodies in** ***RIIB*** ^**−/−**^ **mice. (A)** Anti-ssDNA (*upper*) and -dsDNA (*lower*) antibody levels in sera from female and male *RIIB* ^−/−^ mice, and female B6 mice at different ages. Anti-DNA levels were measured by ELISA and presented as values relative to that in a 12-month-old NZB/W F_1_ control female mouse, whose anti-dsDNA and anti-ssDNA levels were 6.64 μg/ml and 4.69 μg/ml, respectively. Each data point represents the mean value ± SD (*n* ≥3). Significantly different between female *RIIB* ^−/−^ and B6 (*P <0.05) with Student’s *t*-test. **(B–D)** Anti-DNA antibody **(B, D)** levels and ANA levels **(C)** in sera from female and male *RIIB* ^−⁄−^SENDAI or TACONIC mice, *RIIB* ^−⁄−^ *SLAM* ^129^, and B6 mice at different ages. Anti-DNA levels and ANAs were measured by ELISA and HEp-2 staining combined with ELISA, respectively, and presented as values relative to that in a 12-month-old NZB/W F_1_ control female mouse as described in A. Each data point represents the mean value ± SD (*n* ≥3). *P <0.05. Student’s *t*-test. In **B**, the plots for *RIIB* ^−⁄−^ and B6 mice in **A** were superimposed for comparison. In **D**, the plots for *RIIB* ^−⁄−^ *SLAM* ^129^ and B6 mice in **B** and **A**, respectively, were superimposed for comparison.

In addition to determination of the total ANA levels described above, we also measured the anti-DNA levels in sera from mice at different ages by ELISA. Anti-single-stranded (ss)DNA and -double-stranded (ds)DNA antibodies were detected, albeit in very small amounts, in *RIIB*^−/−^ mice at around 12 weeks and 16–20 weeks of age, respectively, and the levels were higher in females than males after 28 weeks of age, while B6 female mice, as healthy controls, at the same ages did not significantly produce anti-DNA antibodies as compared to those in a NZB/W F_1_ mouse as a disease control (Figure 4A). As was the case for the serum ANA levels, *RIIB*^−/−^S*LAM*^129^ mice produced anti-DNA antibodies, particularly abundantly in females (Figure 4B). The bias was not clear in a breeder’s *RIIB*^−/−^ line (Figure 4C, D). These results indicate that our *RIIB*^−/−^ and *SLAM*^129^ mice differently produce and/or maintain ANAs including anti-DNA autoantibodies.

### Weak glomerulonephritis in *RIIB*^−/−^ mice

**Figure 5:**
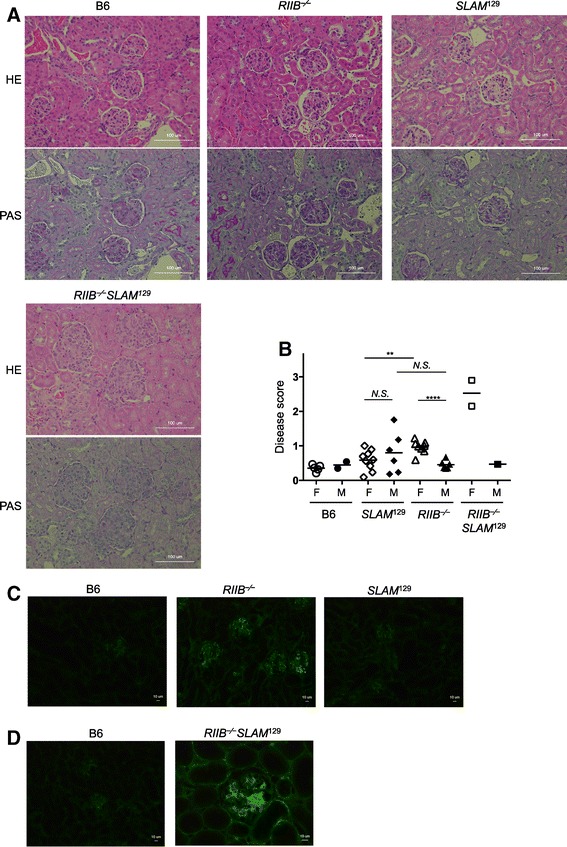
**Glomerulonephritis in** ***RIIB*** ^**−/−**^ **,** ***SLAM*** ^**129**^ **, and** ***RIIB*** ^**−/−**^ ***SLAM*** ^**129**^ **mice. (A)** Kidney sections from each mouse line at week 45 were stained with HE (*upper*) and PAS (*lower*) for examination of glomerular disease, which was assessed according to the criteria depicted in *Methods*. Original magnification, ×200. Scale bar, 100 μm. **(B)** Comparison of the disease scores among *RIIB* ^−/−^, *SLAM* ^129^, *RIIB* ^−/−^ *SLAM* ^129^ and B6 mice at 45 weeks of age. *F*, female; *M*, male. Horizontal bars denote the mean values, excepting the plot of *RIIB* ^−⁄−^ *SLAM* ^129^ male for single determination. ***P <0.001 (*n* =3–6). For scoring, ≥25 glomeruli were examined for each line. Student’s *t*-test. **(C, D)** For IgG-immune complexes (IgG-ICs) deposition, kidney sections from each mouse line at week 45 were stained with FITC-anti mouse IgG. Original magnification, ×200 **(C)** or × 100 **(D)**. Scale bar, 10 μm. The figure is representative of most of the glomeruli observed in three mice per group.

Given the production of anti-DNA antibodies by *RIIB*^−/−^ mice, we next examined the renal histology, and scored the glomerulonephritis in male and female *RIIB*^−/−^, *SLAM*^129^, and *RIIB*^−/−^*SLAM*^129^ mice at 36 weeks of age (Figure 5). While control female *RIIB*^−/−^*SLAM*^129^ mice exhibited severe glomerulonephritis with occasional crescent formation, mesangial cell proliferation, macrophage and neutrophil infiltration, and lobulation of glomeruli, female *RIIB*^−/−^ mice exhibited weak but less severe glomerulonephritis than control *RIIB*^−/−^*SLAM*^129^ animals, with mesangial cell proliferation and neutrophil infiltration (Figure 5A, B). Male *RIIB*^−/−^, and male and female *SLAM*^129^ mice only showed occasional mesangial cell proliferation. We also examined IgG-IC deposition in renal samples by immunofluorescence microscopy, and found that only female *RIIB*^−/−^ and *RIIB*^−/−^*SLAM*^129^ mice exhibited weak deposition (Figure 5C, D), but female *SLAM*^129^ mice did not (Figure 5C). These results indicate that the development of glomerulonephritis observed in *RIIB*^−/−^ mice had occurred in a female-biased manner, while *SLAM*^129^ mice did not develop the disease at least until 36 weeks of age. It is noteworthy that the development of glomerulonephritis seemingly correlated with the female-biased ANA increase in *RIIB*^−/−^ and *RIIB*^−/−^*SLAM*^129^ animals described above (Figure 4A–C), but *SLAM*^129^ mice did not manifest the disease regardless of the fact that they produced ANAs at comparable levels to female *RIIB*^−/−^ mice, at least at 24 weeks of age (Figure 2B, C). This notion suggests a contribution of FcγRIIB to protection from IgG-IC deposition in glomeruli and glomerulonephritis.

### Splenic germinal center formation was grossly normal in *RIIB*^−/−^ mice

**Figure 6:**
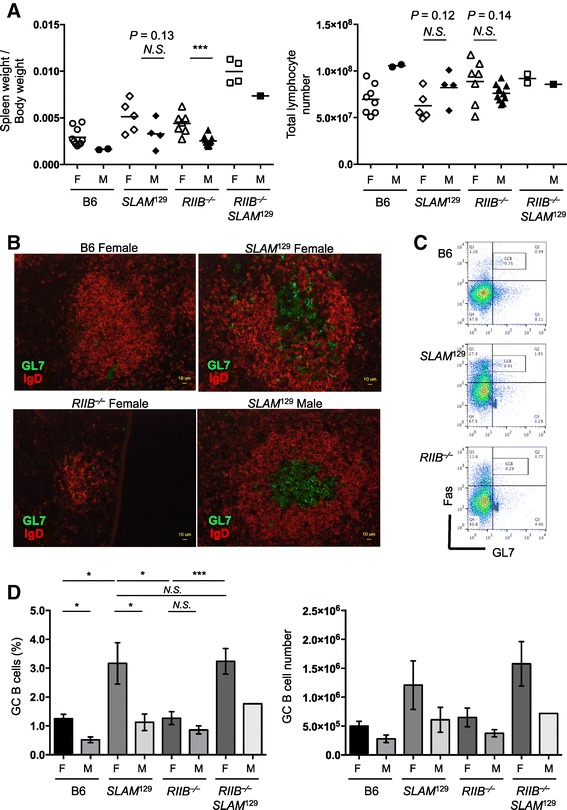
**Germinal center B cells are increased in** ***SLAM*** ^**129**^ **but not** ***RIIB*** ^**−/−**^ **mice.** (**A**, *Left*) Slight splenomegaly in female *RIIB* ^−/−^ mice. Spleen weights were measured for each line of both genders (*F*, female; *M*, male) and are presented as the ratio of the weight per body weight. Horizontal bars indicate mean values, excepting the plot of *RIIB* ^−⁄−^ *SLAM* ^129^ male for single determination. ***P <0.001. Student’s *t*-test. (**A**, *Right*) Total splenic lymphocytes in each line. Horizontal bar represents the mean number of total splenocytes, excepting the plot of *RIIB* ^−⁄−^ *SLAM* ^129^ male for single determination. *N.S.*, not significant. Student’s *t*-test. **(B)** Germinal center formation in naïve B6, N22 female *RIIB* ^−/−^, and female and male *SLAM* ^129^ mice at 45 weeks of age. Frozen spleen sections were stained with GL7-Alexa488 and Alexa546-labeled anti-mouse IgD antibodies. Scale bar =10 μm. **(C)** Flow cytometric determination of germinal center (GC) B cells of B6, female *SLAM* ^129^ and N22 female *RIIB* ^−/−^ mice at 45 week of age. Splenocytes were gated with anti-CD19-APC, and analyzed for anti-Fas-PE and GL7-FITC. **(D)** Total GC cells and GC B cells are not significantly increased in female *RIIB* ^−/−^ mice. The percentage of GC B cells in splenic CD19^+^ cells (*Left*), and the absolute number of splenic GC B cells (*Right*) of each line of both genders (*F*, female; *M*, male) are shown. *P <0.05; ***P <0.001; *N.S.*, not significant. Student’s *t*-test.

We were interested in determining why ANAs and anti-DNA antibodies in sera were increased in *RIIB*^−/−^ mice, albeit in very small amounts. To this end, we isolated spleens, and examined their weights, histology, and cellularity. While splenomegaly was observed in *RIIB*^−/−^*SLAM*^129^ mice, it was much less evident in *RIIB*^−/−^ mice (Figure 6A, *left*). This was, however, not pronounced in the total splenic lymphocytes of *RIIB*^−/−^ animals (Figure 6A, *right*). Immunohistochemistry of spleen sections prepared from naïve mice indicated that germinal center (GC) formation was grossly normal in *RIIB*^−/−^ mice of both genders, while it was augmented in *SLAM*^129^ mice (Figure 6B). The numbers of total GL7^+^Fas^+^ GC cells and CD19^+^GL7^+^Fas^+^ GC B cells were not significantly increased in *RIIB*^−/−^ mice of both genders, while they tended to be increased in female *RIIB*^−/−^*SLAM*^129^ and *SLAM*^129^ animals (Figure 6C, D), these observations being reminiscent of SLAM’s important role in adequate B–T cell communication in GCs [22]. Thus, we failed to find marked alterations in *RIIB*^−/−^ mice in terms of the total splenic lymphocyte and GC cell numbers other than slight splenomegaly. Further analysis of other parameters such as plasma cells, antigen-presenting cells and T cells may help to clarify the reason for the production of a small amount of autoantibodies in *RIIB*^−/−^ mice.

## Discussion

The exact effect of RIIB deficiency on spontaneous development of autoimmune diseases is controversial. Regarding animal models, *RIIB* gene-targeted mice derived from ES cells of the 129 strain and backcrossed into the B6 genetic background yielded different phenotypes in different laboratories, most probably due to different contributions of the autoimmune-prone *SLAM*^129^ haplotype neighboring the *RIIB* locus [4,5,23,24]. In this study, we, for the first time, established two lines through extensive backcrossing, one line with RIIB deficiency but the non-autoimmune B6 *SLAM* haplotype, and the other with autoimmune-prone *SLAM*^129^ and the intact *RIIB* gene, both having the B6 background and being housed under identical environmental conditions. We found that the RIIB deficiency indeed caused a spontaneous increase of a very small amount of ANAs including anti-DNA autoantibodies, and weak glomerulonephritis with slight IgG-IC deposition in glomeruli in a female-biased manner, while the *SLAM*^129^ line exhibited ANA production accompanying no obvious glomerulonephritis, IgG-IC deposition being minimum and comparable to that in the B6 control.

RIIB is known to be an important suppressor of the production and/or accumulation of autoantibodies in some animal models, in which the diseases were induced experimentally in autoimmune-prone mouse strains or non-autoimmune B6 mice by immunization [15,16,25-28]. A recent report [19] has pointed out the additional role of RIIB, expressed in liver sinusoidal endothelial cells, in the clearance of small size IgG-ICs, and potentially in minimizing the level of pathologic IgG-ICs in the blood. In contrast to well-established feedback immunoregulation, and its crucial role in suppressing autoantibody production and/or accumulation after active immunization of susceptible mice, the role of RIIB in physiological suppression of spontaneous autoantibody production in non-autoimmune strains remained unclear due to the inconsistent phenotypes of RIIB-deficient mice observed in different laboratories [8,16-18], the mice being extremely prone to the production of anti-chromatin autoantibodies and susceptible to the development of fatal lupus [8,21], whereas in others they were not susceptible to the development of lupus nephritis without massive production of autoantibodies [16-18]. To circumvent the difficulty in isolating the effect of RIIB deficiency by backcrossing, Boross et al. generated B6- but not 129-origin RIIB-deficient mice by gene targeting in B6-derived ES cells [17]. They observed that RIIB-deficient mice produced only a small amount of anti-ssDNA antibodies spontaneously even at the age of 10 months, exhibited slight IgG-IC deposition in the kidneys, and increases in urinary albumin and the pathological score of glomerulonephritis, without showing mortality at least up to 12 months of age, indicating that RIIB deficiency only amplifies spontaneous autoimmunity determined by other loci. These phenotypes are mostly the same as those of our *RIIB*^−/−^ mice in this study in terms of the very low ANA level and slight IgG-IC deposition without development of glomerulonephritis. Similar results have also been obtained for congenic lines within the *Nba2* autoimmune susceptibility locus [29]. Thus, our present observations substantially support those reported for B6-based *RIIB*^−/−^ mice [17], and our results also indicate that RIIB prevents the spontaneous production and/or accumulation of a small amount of ANAs.

The IgG-IC deposition, albeit slight, and the increase in the pathological score for glomerulonephritis in *RIIB*^−/−^ mice were unexpected, because these phenotypes were not observed in the *SLAM*^129^ line housed under the same environmental conditions as *RIIB*^−/−^ mice (Figure 5). These observations are also consistent with those for the B6-based RIIB-deficient mice [17]. Interestingly, a recent study employing a cell type-specific RIIB deletion technique has shown that RIIB on myeloid cells and intrinsic renal cells rather than B cells prevents nephrotoxic nephritis, suggesting a significant role of RIIB on myeloid cells and renal mesangial cells in this protection [30]. It has also been shown that neither B cell- nor myeloid-specific deletion of RIIB leads to the development of crescentic glomerulonephritis with a higher incidence than in wild-type mice in an anti-glomerular basement membrane antibody disease model, indicating that RIIB deficiency in either B cells or a subset of myeloid cells alone is not sufficient to increase the susceptibility to the kidney disease [31]. In contrast to *RIIB*^−/−^ mice, *SLAM*^129^ animals did not show IgG-IC deposition in the kidneys, even though they had a comparable level of serum ANAs to that in *RIIB*^−/−^ mice, at least at 24 weeks of age. Thus, the *SLAM*^129^ haplotype in the B6 background does not contribute to the development of glomerulonephritis but to ANA production, due to the lack of a significant role in the kidneys.

Generally, development of autoimmune diseases is biased toward females in humans and in mice, the main reason for which being the various influence(s) of sex hormones on the immune system and cells [32-34]. The reason for the gender bias in the ANA levels in *RIIB*^−⁄−^ mice rather than *SLAM*^129^ mice is currently unknown, although one may speculate that antigen-presenting cell–T cell communication could be more prone to be de-regulated in females than B cell–T cell interactions, which could be influenced by *SLAM*^129^. Further examinations will clarify the differences in gender in splenocytes and lymph node cells, and even in those in the lamina propria.

## Conclusion

Separation of the *RIIB*^−/−^ locus from the autoimmune-prone *SLAM*^129^ locus revealed the role of RIIB in maintenance of peripheral tolerance. As judged on assessment under identical genetic and environmental conditions, RIIB deficiency caused slight ANA production and/or its accumulation accompanied by non-lethal glomerulonephritis with a low level of IgG-IC deposition, in contrast to the role of *SLAM*^129^, which causes ANA production without accompanying glomerulonephritis. The combination of the RIIB deficiency and *SLAM*^129^ synergistically induced substantial ANA production and sub-lethal glomerulonephritis. These results will facilitate development of strategies for targeting RIIB for treating autoimmune disorders involving autoantibody production such as SLE.

## Additional file

Additional file 1:
**Tables S1 and S2 and Figures S1 and S2.**

## Abbreviations

ANA: Anti-nuclear antibody
B6: C57BL/6
ds-: Double-stranded
FcγRIIB: Type IIB low affinity Fc receptor for IgG
FcR: Fc receptor
FcγR: Fc receptor for IgG
GC: Germinal center
HE: Hematoxylin and eosin
RIIB: FcγRIIB
*RIIB*^−⁄−^: FcγRIIB-deficient
*RIIB*^−⁄−^SENDAI mice: N28 FcγRIIB-deficient mice
ss-: Single-stranded
SLAM: Signal lymphocyte activation molecule(s)
SLE: Systemic lupus erythematosus
