# Supplementary material for: Dichotomy in FcγRIIB deficiency and autoimmune-prone SLAM haplotype clarifies the roles of the Fc receptor in development of autoantibodies and glomerulonephritis
Source: BMC Immunol. 2014 Oct 24;15:47. doi: 10.1186/s12865-014-0047-y (PMC4209029; doi:10.1186/s12865-014-0047-y)
Supplement: Additional file 1: — Tables S1 and S2 and Figures S1 and S2. [file 12865_2014_47_MOESM1_ESM.pdf]

# Additional File

## Supplementary Table 1

List of PCR primers and sequence primers in SNP analysis

| Gene | Forward Primer | Reverse Primer | Sequence Primer |
|---|---|---|---|
| *AlyRef2* | GTGTCAGATGCTGACATTCAG | ATTGTCTGCACAGTCCAGTTG | TACGATCGCTCTGGACGAAG |
| *Apcs* | GCCAATCTCTGCTACATGAG | GCCAATCTCTGCTACATGAG | GAGCCAAGAGAAAGCGTTTG |
| *Atf6* | CAAGTGGGCCTGGATTCTGCAACAG | GCTTACTTCGCGAGTGCGGCATAAG | GAGCGTTGACTTACCAATAG |
| *Casq* | ACCATCAGAGTCTGGGGAG | ACAGACAGGAAGCAGGGAG | TGTGTGTGTGGCAGGTCCAG |
| *Cd48* | TGGAGGTGGCTGGAGATAG | TACTTCCCCACCTCCCCAG | CCCAGGTCCCGTTTCTCTTG |
| *Cd84* | TTTCCCATTGGCTCGAGGAG | CTGGAACTGATGGGAATGAG | CAAGTTCCCTTGGCTCCTAG |
| *Fcer1a* | ATAAGGACTCTGCTGTGGAG | CTCATCTCCATCTGTTTCAG | GTTAATGGTCATTCATGGATG |
| *Fcer1g* | CTGTGTTCTCAGGAGCTGAG | GATACTCCCTGGGTAGGAAG | CCAAGCTCCACCAGCATGTG |
| *Fcgr3* | CCTCTCCCACGCAAGACTACACAGAG | CCACCACTGCCCAGTACAGTCAG | TGATGCTTCCCAGAAACCAG |
| *Fcrg4* | AGAGTTTGAGGCCAGCCTGGTCTATAAAG | CTCTTGCTGCCAATCCTAACCACCTTAG | TAAAGTTCTAGGACAGCCAGGG |
| *Fcrla* | GAGCCCTCTCGGTAGAAGATAACCTGAG | GAGGTTTGGTGTCTTCTTCTTCTCTCCTGAG | ATATTCCCAGGGCAGAAGAG |
| *Fcrlb* | CCAACAACCAAAACCACAGCAACAGGAG | TCAGTCAGTCACTTCCATCCCGAACAG | GGCTCCAATTTGCACACAAG |
| *Ly9* | CTAGCCAGTGGGCTTAGAAG | CTTGTGGCTTCCATGGCTAG | GAACAACCGCTCATCTCGAG |
| *Mpz* | GGGACATTCTTTATGCTGAGACCTTGAGCAG | CTGTTGGTTGAAGCTATTTGCCCTTCTCAG | CTGAGACCTTGAGCAGATAAAG |
| *Nuf2* | CTTGGTCTAGGGCAGAGTGTTAATACAG | GTCTCTCATGTCATTGTCCTGATTGTCCTGTAG | TGTACCTGGGCAGGGATCAG |
| *Rgs4* | GGCCTATAAAGCACATGGCAGAAGCAG | GTGGTGTTGGGGTTTTCACTAGGGTAAG | CAAATTGGGTCTTACCCTAG |
| *Sh2d1b1* | GAGCAAACATGTCCTTCCTACCTGTGAG<br>TTTGACTTCCTCCCAGACTTGGTCGTAG | GGCCTTTGCACACGCTAAGCAAG<br>GGACAGGCATGGAGATCACCCTGTTAAG | TGTGACCCAACCTGAAACTG<br>TCGTAGCAAATCTTTCCTTG |
| *Slamf6* | GGTACTGCTCTTCTCAGTAG | ACTGCCTGAACAGTGGCAG | AGCCAGACTGCTGCTGAGAG |
| *Slamf7* | GTCTACCTCCCCAGTGCTG | TAGCTCCACCACACACACAG | TGCACTTCTTAAGGGAAGAG |
| *Slamf8* | CAGCCAGGATCAGGAACAG | CAGCAGAGGGTATAACTGAG | TGTAGGAGGCCTTCCCGGAG |
| *Slamf9* | GGTTCTCTGACCTTCAGTAG | CTTGCAACTGGTTGTCCGAG | CTGCTTCTGAGACTCCAAAG |

Supplementary Table 2

Survival rate of mouse lines

| Strain | Survival at 45 wk |
|---|---|
| B6 Female | 17/17 |
| B6.*lpr* Female | 17/17 |
| *RIIB*$^{-/-}$SENDAI Female | 30/30 |
| *RIIB*$^{-/-}$*SLAM*$^{129}$ Male | 18/18 |
| *RIIB*$^{-/-}$TOKYO Male | 4/4 |
| *RIIB*$^{-/-}$TOKYO Female | 7/7 |
| *RIIB*$^{-/-}$*SLAM*$^{129}$ Female | 13/14 |
| *RIIB*$^{-/-}$SENDAI Male | 34/35 |
| *SLAM*$^{129}$ Female | 22/23 |
| *SLAM*$^{129}$ Male | 26/27 |
| *RIIB*$^{-/-}$TACONIC Female | 0/3 |
| *RIIB*$^{-/-}$TACONIC Male | 2/3 |
| *RIIB*$^{-/-}$.*lpr* Female | 0/28 |
| *RIIB*$^{-/-}$.*lpr* Male | 1/12 |

Data for *lpr*-harboring mice are from Yajima K et al. (*Eur J Immunol* 2003, **33**:1020–1029) for comparison.

Supplementary Figure 1

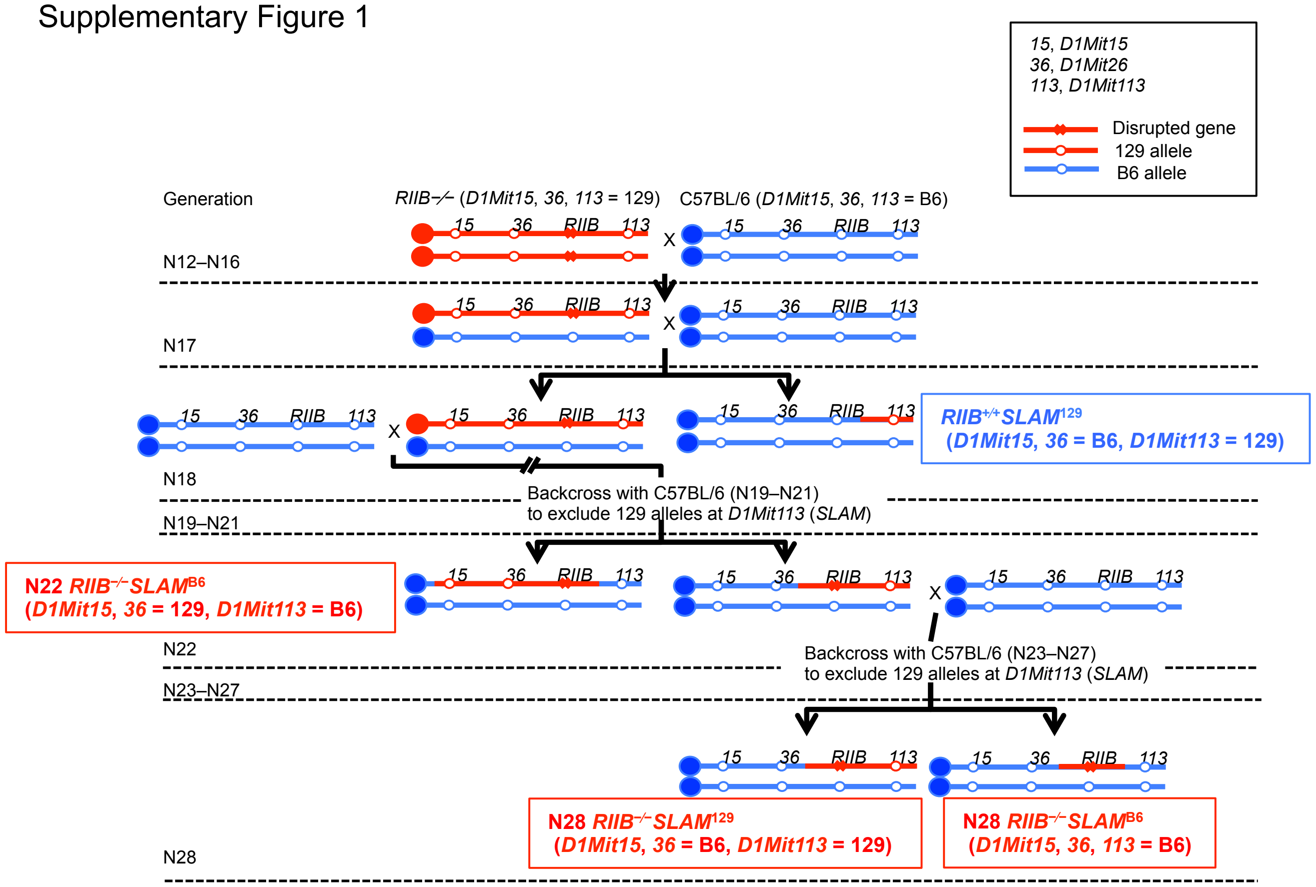


**Supplementary Figure 1. Family tree of *Fcgr2b*-deficient (*RIIB*$^{-/-}$) mice in our laboratory.** The original *RIIB*$^{-/-}$ mice constructed with a 129/B6 hybrid background [13] were backcrossed into the B6 genetic background for 12 generations (N12) as described [16]. At the 18th generation (N18), we obtained *RIIB*$^{+/+}$*SLAM*$^{129}$ heterozygotes, which were intercrossed to obtain the *RIIB*$^{+/+}$*SLAM*$^{129}$ line. At the 22nd (N22) generation, *RIIB*$^{-/-}$*SLAM*$^{B6}$ heterozygotes obtained were intercrossed to generate the N22 *RIIB*$^{-/-}$ line. Further backcrossing of N22 offspring into B6 yielded heterozygotes for *RIIB*$^{-/-}$*SLAM*$^{129}$ and *RIIB*$^{-/-}$ at the 28th generation (N28), each of which was intercrossed to generate homozygotes of N28 *RIIB*$^{-/-}$*SLAM*$^{129}$ and N28 *RIIB*$^{-/-}$*SLAM*$^{B6}$ (*RIIB*$^{-/-}$). Our *RIIB*$^{-/-}$ mouse line is designated as *RIIB*$^{-/-}$SENDAI where it is necessary to distinguish it from those obtained from others. We analyzed the following SSLP markers on chromosome 1: *D1Mit100*, *D1Mit102*, *D1Mit501*, *D1Mit34*, *D1Mit268*, *D1Mit105*, *D1Mit15*, *D1Mit36*, *D1Mit205*, *D1Mit270*, *D1Mit147*, *D1Mit146*, *D1Mit113*, *D1Mit355.2*, *D1Mit150*, *D1Mit221*, and *D1Mit17*.

Supplementary Figure 2

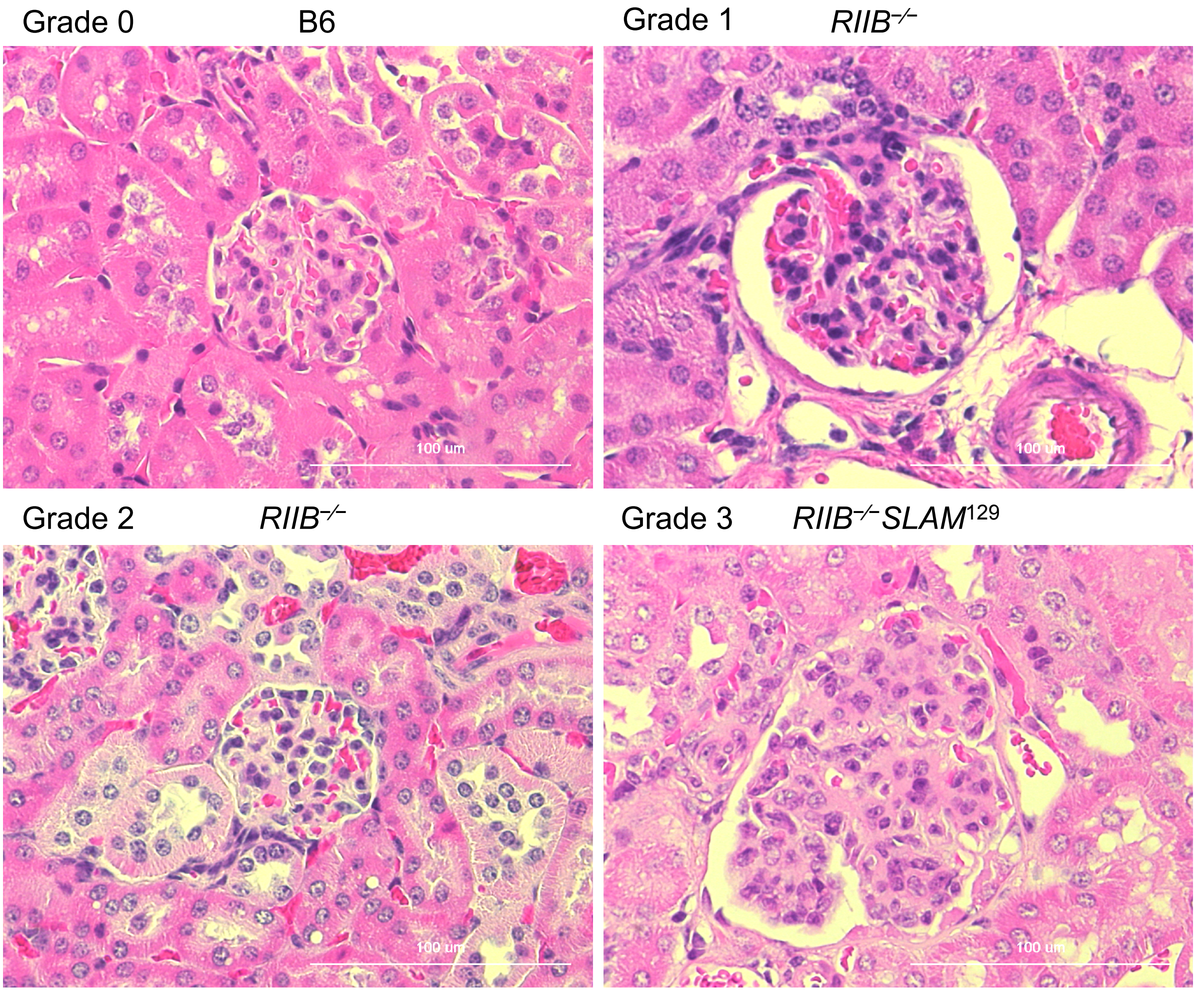


**Supplementary Figure 2. Estimation of the severity of glomerulonephritis.**
The severity of glomerulonephritis was estimated as follows; grade 0, normal; grade 1, neutrophil infiltration and segmental mesangial proliferation; grade 2, limited lobulated glomeruli in grade 1; and grade 3, crescent formation and severe lobulated glomeruli with lymphocyte infiltration. Representative picture for each score is shown with the source of mouse line.
